# Considering Abundance, Affinity, and Binding Site Availability in the NF-κB Target Selection Puzzle

**DOI:** 10.3389/fimmu.2019.00609

**Published:** 2019-03-29

**Authors:** Ruth Brignall, Amy T. Moody, Shibin Mathew, Suzanne Gaudet

**Affiliations:** ^1^Center for Cancer Systems Biology and Department of Cancer Biology, Dana-Farber Cancer Institute, Boston, MA, United States; ^2^Department of Genetics, Harvard Medical School, Blavatnik Institute, Boston, MA, United States; ^3^Laboratory for Systems Pharmacology, Harvard Medical School, Blavatnik Institute, Boston, MA, United States; ^4^Department of Microbiology, Tufts University School of Medicine, Boston, MA, United States

**Keywords:** NF-κB, transcription regulation, specificity, accessibility, competition

## Abstract

The NF-κB transcription regulation system governs a diverse set of responses to various cytokine stimuli. With tools from *in vitro* biochemical characterizations, to omics-based whole genome investigations, great strides have been made in understanding how NF-κB transcription factors control the expression of specific sets of genes. Nonetheless, these efforts have also revealed a very large number of potential binding sites for NF-κB in the human genome, and a puzzle emerges when trying to explain how NF-κB selects from these many binding sites to direct cell-type- and stimulus-specific gene expression patterns. In this review, we surmise that target gene transcription can broadly be thought of as a function of the nuclear *abundance* of the various NF-κB dimers, the *affinity* of NF-κB dimers for the regulatory sequence and the *availability* of this regulatory site. We use this framework to place quantitative information that has been gathered about the NF-κB transcription regulation system into context and thus consider questions it answers, and questions it raises. We end with a brief discussion of some of the future prospects that new approaches could bring to our understanding of how NF-κB transcription factors orchestrate diverse responses in different biological contexts.

## Introduction

**Figure 1:**
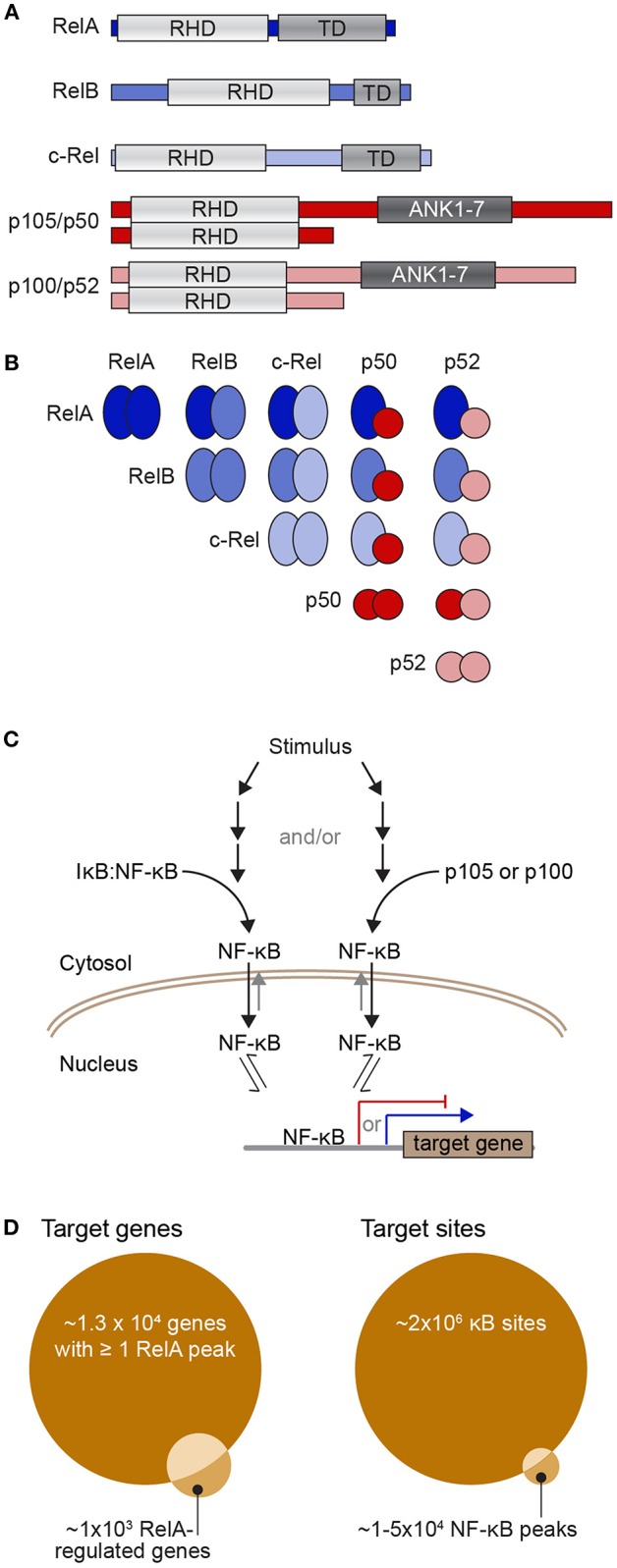
The NF-κB transcription factor family and its dimerization and DNA interactions. **(A)** Diagrams of the five NF-κB subunits showing their Rel homology domain (RHD), which encompasses both their DNA-binding domain and dimerization region, the transactivation domains (TD) of RelA, RelB, and c-Rel, as well as the ankyrin-rich region of p105 and p100 (repeats 1-7; ANK1-7), which is cleaved to yield p50 and p52. **(B)** Diagram of the ways in which the NF-κB subunits can partner to form dimers that contain zero (all red), one (blue/red), or two (all blue) transactivation domains. **(C)** Simple schematic of the process of activation of NF-κB dimers. Upon stimulation, a series of events leads to phosphorylation and proteasome-mediated degradation of IκB to release NF-κB dimers (left) and/or cleavage of p105 or p100 to remove their IκB-like ankyrin-rich domain and again release NF-κB dimers (right). Free NF-κB dimers are preferentially shuttled into the nucleus where they have access to the regulatory sequences of NF-κB target genes. TD-containing NF-κB dimers can activate transcription of target genes (blue arrow), while TD-lacking NF-κB dimers can act as transcriptional repressors (red). **(D)** Venn diagrams representing potential target sites (right) and potential target genes (left). There may be up to 2 × 10^6^ consensus κB sites or half-sites in the human genome (1) although various ChIP-seq studies have reported that there may be between 1 and 5 × 10^4^ NF-κB-bound peaks in a mammalian genome across a population of stimulated cells [e.g., (2–4)], of which 30–50% contain a consensus κB site. Because many gene regulatory sequences have multiple NF-κB-bound peaks, one estimate is that around 1.3 × 10^4^ genes have at least one RelA peak in their regulatory region (5). However, the same study found only ~1,000 genes were detectably regulated by RelA-containing NF-κB with ~60% of these having a RelA ChIP-seq peak in gene-proximal regulatory regions (5).

The nuclear factor-κB (NF-κB) family of transcription factors regulate the expression of genes that are crucial to a wide variety of biological processes, ranging from immune, stress, and inflammatory responses, to cell apoptosis. The NF-κB family is made up of five proteins, p105/p50 (encoded by *NFKB1*), p100/p52 (encoded by *NFKB2*), RelA (also known as p65), RelB, and c-Rel, which can form a range of homo- and hetero-dimeric complexes [Figures 1A,B; (6)]. When partnered with inhibitory IκB proteins, NF-κB dimers are preferentially shuttled to the cytoplasm where they are held inactive. In response to stimuli, IκB is phosphorylated and subsequently degraded, thus releasing NF-κB and allowing it to accumulate in the nucleus (Figure 1C). Once in the nucleus an NF-κB dimer can bind to κB sites to activate or repress the transcription of its target genes. The best-studied κB sites fit the consensus κB site pattern, 5′-GGGRNWYYCC-3′ (where R, W, Y, and N, respectively denote purine, adenine or thymine, pyrimidine, and any nucleotide) (7–9). In the human genome encompassing 3 × 10^9^ base pairs, there are undoubtedly myriads of sequences matching the consensus κB site. Indeed, early on, ChIP-chip (chromatin immunoprecipitation to microarray) experiments interrogating the sequence of human chromosome 22 suggested that there are more than 1.4 × 10^4^ of these consensus sites contacted by NF-κB dimers during a response to stimulus (2, 10). More recent ChIP-seq experiments have identified 20,000–50,000 RelA-bound peaks, although it is unclear whether RelA directly contacts the DNA at all of these sites (3, 4). Just considering the RelA subunit, 1 × 10^5^-1.5 × 10^5^ molecules enter the nucleus following stimulation (11), a portion of these molecules bind to DNA and this regulates the expression of just ~600 genes [a curated list of known NF-κB target genes can be found at the Boston University NF-κB Transcription Factors website; (12)]. The large number of RelA molecules in comparison to the relatively small number of regulated transcripts suggests a complex relationship between the amount of NF-κB in the nucleus and the subsequent expression of target genes.

Numerous ChIP-seq and whole genome sequencing experiments have shown that the recruitment of many transcription factors, including NF-κB, to chromatinized DNA is dependent on the cellular context and therefore must be highly regulated [reviewed in (10)]. However, despite the wealth of genomic data now available, the mechanisms by which NF-κB-DNA interactions generate specific gene expression profiles following stimulation remain largely unknown. For example, by allocating ChIP-Seq peaks to their nearest gene, RelA-containing NF-κB was found to bind ~13,600 genes in TNF-stimulated HeLa cells, yet only ~1,000 genes were up or down-regulated in response to RelA perturbation, and only ~600 of these were directly bound by NF-κB [Figure 1D, left; (5)]. Thus, a vast majority of the genes that are bound by NF-κB in response to stimulus are not regulated. This raises the questions: how do NF-κB dimers select their binding sites and why are only some of the bound genes transcriptionally regulated? Seeking to answer these questions, we will focus herein on three key sets of factors that regulate NF-κB recruitment to DNA: *abundance* of NF-κB dimers and κB binding sites, binding *affinity*, and the *availability* of the κB sites at any given time.

## Abundance

### κB Binding Sites

If, as Martone et al. (2) estimated, there are ~10^4^ consensus κB sites in the genome that are bound by RelA and ~1 × 10^5^ RelA-containing dimers enter the nucleus upon cell stimulation [estimated by Hottiger et al. (11)], a simple view of the system would predict rapid saturation of these consensus κB sites (see Box 1). However, experiments demonstrate that many consensus κB sites are not bound and, in fact, this lack of saturation of the system is necessary to generate stimulus- and cell-type-specific gene expression profiles (16–18). One explanation for this apparent dichotomy is that, in addition to consensus κB sites, NF-κB can bind to degenerate κB sites. Structural, biochemical, and *in vivo* assays have demonstrated that NF-κB dimers can bind to κB half sites, sites whose sequences deviate from the consensus sequence, and even unrelated sites (3, 19–24). With these additional non-consensus binding sites, the total number of potential NF-κB sites in the human genome could easily climb to 2 × 10^6^ (1). This flips the NF-κB protein vs. NF-κB binding site calculus (Figure 1D, right), and our first question becomes: how do the relatively *sparse* NF-κB dimers decide which of the *numerous* potential κB binding sites to interact with?

Box 1 **Computing fraction of binding sites occupied by transcription factor.**

Computational models provide a powerful means to examine, interrogate, and ultimately better understand the relationships between inputs and outputs of complicated biological processes. Here, we use a simple mass-action kinetics model to illustrate how (i) binding affinity, (ii) abundance of transcription factors and their binding sites, and (iii) the availability of these binding sites due to the presence or absence of a binding competitor species affect the fraction of sites bound by the transcription factor. Although in reality, binding by a transcription factor is only a rough correlate of gene transcription in response to stimuli, this toy model shows us how the interplay between quantitative aspects of protein-DNA interactions potentially affects transcription regulation. Previous studies have used similar kinetics models to calculate fraction of binding sites (13).

In the simple scenario that we depict (Figure Box 1A), we model the binding of transcription factors to their cognate sites on the genome as a simple adsorption process—where molecules bind to sites, unchanged. This model therefore gives us a theoretical limit on the fraction of bound sites when the process is activation energy-limited (i.e., within-nucleus transport is much faster than DNA binding) and the process of a transcription factor finding a binding site is random. We also make additional simplifying assumptions: (1) the contents of the nucleus are well mixed and both genomic and non-genomic compartments are homogeneous; (2) all binding sites are equivalent with identical affinities for the transcription factor and competitor species; (3) the total nuclear concentrations of transcription factor and competitor species are fixed, under the assumption that any change occurs on a time scale slower than that of the binding process (and therefore, in this very simplistic model, we assume that the steady state is reached faster than changes in nuclear abundance and post-translational modifications of transcription factors). Given the stated assumptions, we will let *X* be free nuclear transcription factor, *X*^*comp*^ be free nuclear competitor species, and *Y* represent the transcription factor binding site. *Y* can be bound by *X* or *X*^*comp*^ creating the complexes *Y*:*X* and *Y*:*X*^*comp*^, respectively. Binding of transcription factor and competitor species to DNA can then be modeled by two reaction equations:

$$\begin{array}{l} X+Y\;↔\;Y:X\\ X^{comp}+Y\;↔\;{Y:X}^{comp} \end{array}$$

As we assumed identical affinities to DNA binding sites for the transcription factor and its competitor, we will also assume the same association rate parameter α and dissociation rate parameter γ. Using mass action kinetics and mass balance equations, our reaction system can be fully described using two ordinary differential equations (ODEs):

$$\begin{array}{l} \frac{d\left[Y:X\right]}{dt}=\alpha \cdot \left(X_{T}-\left[Y:X\right]\right)\cdot \left(Y_{T}-\left[Y:X\right]-\left[{Y:X}^{comp}\right]\right)-\gamma \cdot \left[Y:X\right]\\ \frac{d\left[{Y:X}^{comp}\right]}{dt}=\alpha \cdot \left(X_{T}^{comp}-\left[{Y:X}^{comp}\right]\right)\cdot \left(Y_{T}-\left[Y:X\right]-\left[Y:X^{comp}\right]\right)-\gamma \cdot \left[{Y:X}^{comp}\right] \end{array}$$

Here, *X*_*T*_, $X_{T}^{comp}$ and *Y*_*T*_ are the total number of molecules or sites for a given nucleus and, under our assumption of time scale separation (#3), they are assumed constant while solving the ODEs. We also define $K_{D}=\;\frac{\gamma }{\alpha }$, the dissociation constant (which is, as usual, the inverse of the binding affinity constant). Solving the system of ODEs gives the concentration of each species over time and at steady state. Solving the ODEs for different sets of parameter values and initial concentrations for *X*_*T*_, $X_{T}^{comp}$ and *Y*_*T*_ allows us to illustrate the relationships between these parameters and initial concentrations and the steady state (ss) fraction of sites bound by the transcription factor, calculated as $\frac{{\left[Y:X\right]}_{ss}}{Y_{T}}$.

To survey a biologically relevant range of concentration values, we considered a typical HeLa cell, with a total cell volume of 2,700 μm^3^, a cytoplasmic to nuclear volume ratio of 3.3 (14), and ratios of transcription factors and available binding sites from 10^3^:10^6^ to 10^6^:10^3^ molecules/site. In the absence of the competitor (Figure Box 1B; continuous lines) and for *K*_*D*_ > 10 nM, we need a large amount of transcription factor ($\log_{10}\left[\frac{X_{T}}{Y}\right]>1$) to effectively saturate most of the binding sites at steady state. If we consider the RelA:p50 heterodimer, which has been reported to bind to the consensus κB site with a *K*_*D*_ of 12.8 ± 2.2 nM (15), and ~1.5 × 10^5^ heterodimers in a nucleus (11) with ~1.4 × 10^4^ binding sites (2), we obtain $\log_{10}\left[\frac{X_{T}}{Y_{T}}\right]>1$, and the simple model finds that >90% of the DNA binding sites would be occupied. In the presence of the competitor species (in abundance equal to that of the dimers) the achievable occupancy is reduced to half (Figure Box 1B, dashed lines). Even a simplistic illustrative model such as the one we used here clearly shows how the interplay between abundance and affinity changes the fraction of bound sites in a nonlinear fashion, and how one mechanism for regulating availability of the sites—competition—can substantially reduce the number of sites occupied by a transcription factor. As we discuss in this review, there are many other nuances to abundance, affinity, and availability which will require more complex models to fully capture.

**Figure Box 1:**
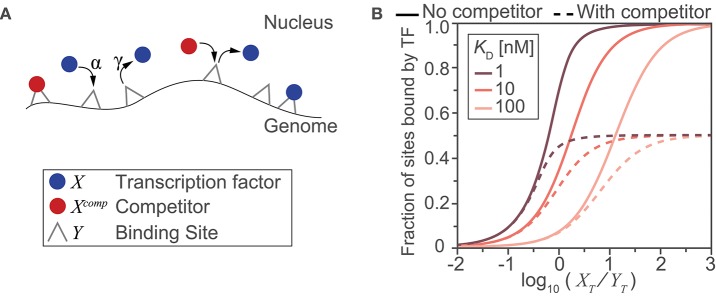
A toy model illustrates the impact of abundance, affinity and availability on the fraction of potential sites bound. **(A)** Schematic diagram of the reactions and molecular species included in the model. DNA binding sites are present on the genome and can be bound by a transcription factor (blue) or by a competitor protein (red) with an on-rate of α and off-rate of γ. **(B)** Model-derived input-output relationships between the ratio of total transcription factor to potential binding sites ($\frac{X_{T}}{Y_{T}}$) and the steady state fraction of sites that are bound by a transcription factor. The relationship was derived for three different transcription factor binding affinities for the DNA sites (expressed using the dissociation constant, $K_{D}=\;\frac{\gamma }{\alpha }$), in the presence (dashed lines) or absence (solid lines) of a competitor species (where, $X_{T}=X_{T}^{comp}$).

In recent years, innovative live-cell imaging techniques based on fluorescence recovery after photobleaching (FRAP), along with kinetic modeling of the collected data, have started to shed light on the dynamic nature of the transcription factor-DNA interaction process. Broadly speaking, this work indicates that most transcription factors may rapidly diffuse through the nucleus (with diffusion coefficients of ~0.5–5 μm^2^s^−1^ depending on transcription factor size) while “scanning” the genome for high-specificity sites (25, 26). Of note, the use of the term “scanning” should not necessarily evoke the image of a transcription factor gliding along chromatin, although such one-dimensional sliding models have been posited following single-molecule imaging studies of the p53 transcription factor (27, 28). Instead, many transcription factors, including NF-κB dimers, may “scan” by visiting multiple sites in a trial-and-error series of short-duration binding events (29). Therefore, transcription factors undergo thousands of these transient encounters with chromatin that ultimately will have no direct consequence on gene expression.

Interestingly, it is now thought that most *functional* NF-κB interactions with chromatin—interactions that lead to a change in transcription—are fleeting. Early, *in vitro*, bulk biochemical measurements of NF-κB interactions with κB sites indicated the formation of very stable complexes with a half-life of up to 45 min (30); using bulk, ChIP-based assays, similarly long interaction half-lives have been measured for other transcription factors (31) and shown to be regulated by ubiquitylation [reviewed in (32, 33)]. However, a subsequent study using FRAP in live cells expressing RelA-GFP showed that most RelA-DNA interactions are actually quite dynamic, with half-lives of a few seconds (16). Using stopped-flow kinetics and surface plasmon resonance, which can both directly measure association and dissociation kinetics, *in vitro* measurements made in physiological salt and pH conditions recapitulated these faster kinetics [yielding half-lives of 1.5 and 40 s, respectively for RelA:p50-DNA (34) and a range of a few seconds to a few minutes for a variety of NF-κB dimers and binding sites (22)]. Strikingly, IκBα can further accelerate the RelA:p50-DNA dissociation by up to ~40-fold, “stripping” dimers from DNA in a process that has now been extensively characterized (34–39). Indeed, a recent study used single-molecule tracking of individual Halo-tagged RelA molecules in live cells to show that the majority (~96%) of RelA undergoes short-lived interactions lasting on average ~0.5 s, while just ~4% of RelA molecules form more stable complexes with a lifetime of ~4 s (40). Because the ability of the RelA fusion proteins to induce transcription of target genes was verified in both the FRAP and single-molecule *in vivo* studies, these results suggest that long-lasting NF-κB binding may not be required for preinitiation complex assembly or for the activation of transcription. However, the mechanisms that distinguish NF-κB-DNA binding events that change transcription of a target gene from those that do not remain unclear.

Recent studies have found that while individual interactions are very brief, the integrated target site occupancy of Sox2 and Oct4 transcription factors can be highly sensitive to the nuclear concentration of the transcription factor (41). This implies that even when transcription factor occupancy at target sites is short-lived, high nuclear concentrations facilitate rapid turnover and, overall, increase the frequency of these short interactions. In this way, high rates of transcription factor sampling at binding sites may generate enough cumulative site occupancy to affect transcription (29). Having many binding sites across the human genome, NF-κB may also use this mechanism to tune the spatiotemporal patterns of gene expression it generates in response to stimulation by, for example, having a larger effect on sites that have the highest cumulative occupancy.

Intriguingly, high frequencies of transcription factor sampling have also been observed at non-consensus sites, yet these interactions were shown to have no direct effect on transcription (42). This observation has revived ideas first proposed years ago, whereby a key part of the target search process is transcription factors making non-specific contacts with DNA and then proceeding to slide or hop around the local chromatin environment until either a specific contact is formed, or contact and access to DNA is lost (43, 44). This model could partially explain the large number of sites detected by static, end-point biochemical binding assays including ChIP-Seq studies, which appear to be non-functional. Although these sites are “visited” in the search process, their cumulative occupancy may not be sufficiently long, or their interaction qualitatively suitable, to affect transcription.

If transcription factors rapidly sample many sites, would a cluster of non-functional binding sites near a target gene promoter or enhancer increases, or decreases, the local concentration of transcription factor? An increase in local concentration could occur if the brief interactions with clustered sites keep more transcription factor molecules nearby, increasing the probability that one binds to the functional target site [e.g., via an avidity effect as theoretically considered by (45)]. Alternatively, if the non-functional sites sequester transcription factors away from the functional target sites, acting as “natural decoys” (46), they would effectively decrease the local concentration of transcription factors. An early study showed that transfection of double-stranded oligonucleotides with κB sites inhibits the NF-κB-induced production of inflammatory cytokines in a rat model of myocardial infarction (47) showing a decoy-site effect. However, in that scenario, the transfected oligonucleotides likely reduce the global, not local, concentration of available NF-κB dimers and thus globally suppress transcription NF-κB-driven transcription. A more direct test of the effects of clusters of sites would be to manipulate the sequences near an NF-κB target gene promoter. In budding yeast, synthetic promoters were used to show that adding clustered sites for a transcriptional activator reduce the transcriptional output, as expected for decoys (48). In addition, the clustered sites could qualitatively change a transcriptional response from a graded response, correlated to transcription factor abundance, to a threshold-based, non-linear response (48). A combination of mathematical models and synthetic *LacI*-based constructs in *E. coli* showed that varying the number and chromosomal context of repressor binding sites can also quantitatively and qualitatively change the response (49). With so many possible nuances driving up or down the probability of transcription factor-DNA interactions, it may well be that the effect of additional sites on the transcription of an NF-κB target gene, whether enhancing or dampening, is highly context-dependent. The relative affinity, number, and two- or three-dimensional clustering of the sites could all modulate their effects and diversify the response of target genes to one NF-κB signal.

In one more layer of complexity, many NF-κB target genes have multiple κB sites *within* their regulatory regions [a common feature of many transcription factor binding motifs; (50)]. In fact, 95% of up-regulated and 91% of down-regulated NF-κB target genes have been shown to contain more than three κB sites in their regulatory regions [e.g., (2, 5)]. For many years, the predominant model for transcriptional regulation was that having multiple sites within gene promoters would drive cooperativity in DNA binding by the cognate transcription factors (51). This cooperative binding was then thought to lead to rapid, binary switching between fully unoccupied inactive promoters and fully occupied active promoters, yielding a largely all-or-none transcriptional activation (or repression) response. However, more recent studies have shown that NF-κB (via RelA) does not generally show cooperative binding to DNA, and instead NF-κB-dependent transcriptional activity scales gradually with NF-κB nuclear concentration (52). Therefore, Giorgetti and colleagues propose that the presence of multiple κB sites in one regulatory region increases the dynamic range of transcriptional outputs, with promoters with more consensus κB sites driving higher transcription at the same nuclear concentration of NF-κB, thus providing yet another means to quantitatively modulate NF-κB-dependent gene expression.

In summary, these observations indicate that the distribution of κB sites in the genome is non-uniform and clustering of the consensus and non-consensus sites in combination with highly frequent interactions of NF-κB with these sites can influence the transcriptional logic as well as shape the dynamic range of transcription. As, in fact, most human transcription factors are generally observed to bind to only a fraction of their consensus sites in any given cell type (53); this site selection process may be a generalized mechanism to achieve specific transcriptional responses.

### Nucleus-Localized NF-κB Dimers

**Figure 2:**
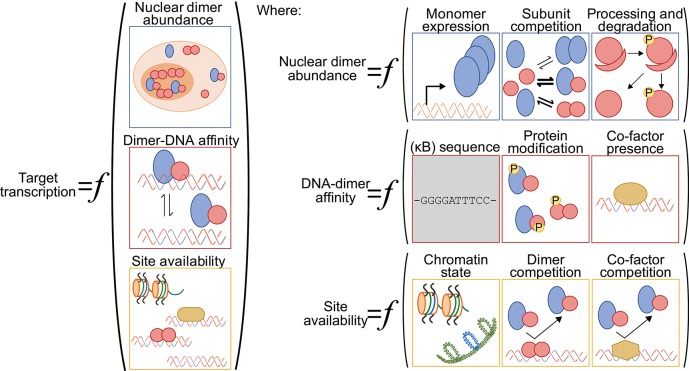
A generalized function for NF-κB-driven gene regulation. Schematic diagram of quantitative and qualitative factors that can differentially modulate NF-κB-driven gene regulation gene-by-gene and across various cellular contexts. Broadly speaking, target gene transcription is set by the nuclear abundance of NF-κB dimers, the NF-κB-DNA binding affinity and the availability of the DNA binding sites (left). The nuclear abundance of NF-κB dimers is itself a function of NF-κB subunit monomer expression, of NF-κB subunit competition in the various dimerization reactions, and of processing and degradation of inhibitory domains (ankyrin-rich domains of p105 and p100) and inhibitory proteins (IκBs) (top right panel). The NF-κB-DNA binding affinity is influenced by the DNA sequence (for both consensus κB sites and non-consensus sites), by NF-κB dimer identity and their post-translation modifications, and by the presence of regulatory co-factors that may help recruit NF-κB dimers to DNA or stabilize the interactions (center right panel). We note here that the DNA sequence is, arguably, the only factor that is not cell-type specific (gray shading). Finally, the availability of DNA binding sites for interaction with an NF-κB dimer is a function of the chromatin state, including the presence of histones and histone modifications, of competition from other NF-κB dimers and their relative affinities for the same site, and of competition with other regulatory factors that may bind to and occlude the potential binding site (bottom right panel).

One challenge for anyone surveying the NF-κB literature with a quantitative mindset is that the terms “NF-κB” and “RelA” are often used interchangeably, and most studies reporting on the abundance of “NF-κB” in the nucleus focus exclusively on the RelA subunit. By ignoring other dimer species, these numbers muddle the relationship between signal, nuclear “NF-κB,” and DNA binding or transcription output. Different stimuli can lead to the nuclear accumulation of specific NF-κB dimers, indicating the importance of considering more than just the RelA subunit [e.g., (54)]. As other reviews have considered stimulus-specific activation of particular NF-κB dimers (17, 55); here, we specifically consider how the nuclear abundance of different dimer species can modulate NF-κB-driven transcriptional responses (Figure 2).

The five NF-κB subunits can dimerize in almost every combination, each with unique but overlapping DNA and protein binding affinities [Figure 1B; (3, 22, 23, 56)]. RelA, RelB, and c-Rel each contain a transactivation domain (TD), capable of recruiting the transcription machinery, and thus NF-κB dimers including at least one of these subunits can activate transcription. In contrast, p50 and p52 do not have a TD and homodimers or heterodimers made up of only p50 and p52 are not capable of inducing transcription without recruiting an additional TD-containing transcription factor. Bound to the same κB site, a TD-containing NF-κB dimer will likely act as a transcriptional activator while a TD-lacking NF-κB dimer can act as a transcriptional repressor.

Although RelA:p50 is frequently cited as the most abundant NF-κB dimer, this may be dependent on cellular context. Other dimer species can also be highly expressed, and some are more likely to be found in the nucleus prior to stimulation. For example, p50 homodimers localize to the nucleus in resting mouse bone marrow derived macrophages (BMDMs) at a concentration of ~200 nM, which is similar to the maximum nuclear RelA:p50 concentration following stimulation in these cells (57). Thus, a quantitative framework that seeks to explain or predict NF-κB-DNA interactions and NF-κB-driven transcription at target genes but considers only RelA:p50 dimers is greatly oversimplifying the system. The 1.5 × 10^5^ molecule per cell figure that we have considered overlooks contributions from other dimer species, the nuclear concentration of which is not necessarily correlated with that of RelA:p50. Therefore, the simple assumption that nuclear RelA:p50 is the major contributor to NF-κB-driven transcription not only underestimates total nuclear NF-κB abundance, but may also obscure the true relationship between “NF-κB” abundance and DNA-binding and transcription activation (see also Box 1).

Finally, the abundance of the different NF-κB dimers is not a static quantity. Some stimuli induce the production of specific dimer species, for example via processing of the p100 subunit to p52, which leads to an increase in p52-containing dimers [Figures 1C, 2; (55)]. There is also competition between the various NF-κB subunits for dimerization, due to the similar affinity of multiple subunits for a given subunit dimerization partner [Figure 2; (58)]. Therefore, if, for example, p52 abundance increases, not only could this induce an increase in repressive p52:p52 dimers, competition for NF-κB dimerization will reduce the abundance of lower dimerization affinity subunit pairs, which could lead to splitting of dimers containing two TD domains to generate p52-containing heterodimers, and effectively increase the abundance of transcription activating NF-κB dimers. Overall, although many studies consider only one protein, the RelA subunit, the total nuclear abundance of NF-κB factors could be substantially higher and the relative abundances of various dimers dynamically modulated. In the section ‘Competition between NF-κB dimers' below, we come back to this and discuss how different dimer abundances can impinge on κB binding site availability.

As we add resolution to quantitative understanding and models of NF-κB-driven transcription in various cellular contexts, we will need to reevaluate simplifying assumptions about the abundance of NF-κB dimers and consider the contributions of the combinatorial possibilities of the “NF-κB dimer network” (17). Because of dimer-specific activities, transcription is certainly impacted by subunit abundance and competition for partnering with TD-containing subunits.

## Affinity of NF-κB Dimers for κB Binding Sequences

Biochemical DNA binding studies of a wide variety of 9–12 base-pair sequences have revealed that different NF-κB dimers bind far more sequences than previously thought, with different dimer species exhibiting specific but overlapping affinities for consensus and non-consensus κB site sequences (3, 22, 23). Although specific NF-κB dimer-DNA affinity values are hard to pin down because they are strongly condition-dependent (15), a constant is that for a given sequence and assay, the affinities of different dimers are consistent with more than one dimer being able to bind this sequence in cells [e.g.,(15, 22, 34)]. Many sequences that contain only a single consensus half-site also show substantial dimer binding (22). Furthermore, structural studies showed that in certain conformations, only one subunit of NF-κB dimers is involved in sequence-specific DNA interactions (24). Taken together, these studies indicate that κB half sites are sufficient for functional NF-κB dimer binding and that the state of the dimer may direct its binding toward certain sequences. Importantly, just as dimers exhibit preferences for different DNA sequences (Figure 2), the corollary must be true, that different DNA sequences may recruit one specific dimer combination over another.

Interestingly, once bound to DNA, each NF-κB dimer has been shown to induce different amounts of transcriptional activity from target genes [reviewed in (56)]. The clearest example, as mentioned above, is that because neither p50 or p52 possesses a TD, dimers containing just these subunits are unable to activate transcription alone. More subtle differences have also been reported, for example, the decreased recruitment of RNA polymerase II (RNAPII) as the *IL12B* promoter switches from binding RelA-containing dimers to RelB-containing dimers (59). The combination of dimer specificity with dimer switching during a response can thus provide a mechanism to generate temporally diverse NF-κB-dependent transcription responses. On the one hand, a response could be abbreviated when TD-containing dimers driving transcription are replaced with TD-lacking repressing dimers, to switch off gene transcription. In a specific example, the stabilization of p50 homodimers during the response of macrophages to LPS stimulation leads to curtailing of the pro-inflammatory transcription of *TNFA* (60), likely via a switch from transcriptionally active dimers to inactive p50 homodimers at the promoter region. By contrast, a switch to the p52/RelB heterodimer, which is insensitive to inhibitory IκB proteins, was found to facilitate the sustained activation of target genes such as *NFKBIA* and *NFKB2* [encoding IκBα and p100/p52, respectively; (59)]. Therefore, the intricacies of sequence-specific affinities of NF-κB dimers and dimer-specific RNAPII-recruiting activities can enable not only tuning of the strength but also the duration and temporal patterns of transcriptional responses at target gene promoters.

How might different consensus κB sites modulate the activity of the NF-κB dimers? Structure-function studies have shown that binding to different consensus κB sites can alter the conformation of the bound NF-κB dimers, thus dictating dimer function [(61, 62), reviewed in (10, 63)]. When an NF-κB dimer interacts with a DNA sequence, side chains of the amino acids located in the DNA-binding domains of dimers contact the bases exposed in the groove of the DNA. For different consensus κB site sequences different bases are exposed in this groove, and NF-κB seems to alter its conformation to maximize interactions with the DNA and maintain high binding affinity (61). Changes in conformation may in turn impact NF-κB binding to co-regulators of transcription, whether these are activating or inhibitory, to specify the strength and dynamics of the transcriptional response (64). These findings again highlight how the huge array of κB binding site sequences must play a key role in modulating the transcription of target genes.

Finally, as an additional layer of dimer and sequence-specific regulation, each of the subunits can be phosphorylated at multiple sites with, depending on the site, effects on nearly every step of NF-κB activation [reviewed in (55)]. While the function of each phosphorylation site is still emerging, there are clear examples of phosphorylation events that have κB-sequence-specific effects on DNA binding and transcription (Figure 2). One of these is the phosphorylation of serine 329 (Ser329) of p50. This phosphorylation attenuates the affinity of p50 for consensus κB sites with a cytosine (C) vs. adenosine (A) at position −1, leading to differential transcriptional activation at A- vs. C-containing sites (65). In addition to effects on NF-κB dimer affinity for DNA, we note that phosphorylation at other sites on the NF-κB subunits has also been shown to affect dimer abundances, via effects on dimerization, monomer and dimer stability, affinity of IκBs, and nuclear translocation rates [reviewed in (55)].

Overall, when considering the various ways in which NF-κB dimer abundances and their affinity for DNA can be modulated, it becomes clear that with these multiple cascading effects, small differences in consensus κB site sequences and small *a priori* differences in interaction affinities can ultimately have a large impact on the transcriptional response to NF-κB pathway activation.

## Availability of High Affinity κB Binding Sequences

### Chromatin State

So far, in our discussion of the large numbers of κB sites on DNA and the high nuclear abundance of NF-κB dimers upon stimulation, we made a strong implicit assumption that all the consensus κB sites and half sites are available for binding. Given their high abundance, nuclear NF-κB dimers should be able to locate and bind to many consensus κB sites and half sites within minutes of an initial cell stimulation. However, ChIP-PCR studies in the murine monocytic cell line Raw 264.7, have shown that while recruitment of NF-κB occurs rapidly after LPS addition for a subset of genes (e.g., *NFKBIA* and *CXCL2*), other gene promoters containing high affinity κB sites remain unbound by NF-κB dimers for over an hour (e.g., *CCL5* and *IL6*) despite the continued presence of nuclear NF-κB dimers (66). This kinetic complexity of the recruitment of NF-κB dimers to DNA during a stimulus-induced response has been largely attributed to variable, chromatin-state-dependent accessibility following stimulation.

The promoter regions of early response genes have abundant histone acetylation or trimethylation prior to stimulation [e.g., H3K27ac, (67) and H4K20me3, (66)], a chromatin state “poised” for immediate activation. This chromatin state may lead to a more open chromatin structure, constitutively accessible to transcription factor binding (66, 67). In contrast, promoters of late genes often have hypo-acetylated histones, requiring conformational changes to the chromatin to become accessible. They are therefore unable to recruit NF-κB for up to several hours after stimulation (68), due to the slow process of chromatin remodeling. Of note, we recently observed that recruitment of RelA-containing dimers displayed similar rapid binding kinetics at highly and poorly acetylated H3 HIV LTR insertions, but recruitment of RNAPII was different, with highly acetylated H3 correlating with more efficient transcription (69). Others have also reported early appearance of nascent transcripts of late genes, again hinting that, at least in some contexts, recruitment of NF-κB dimers may take place early, but that differential stability or processing of the transcript leads them to accumulating only later (70–72). Nevertheless, taken together, these different studies of chromatin state and NF-κB dimer binding suggest that despite the large repertoire of potential binding sites, only a fraction of these sites are available for binding, or for active recruitment of RNAPII, at any given time. This accessible repertoire can change upon stimulation and is dictated by the epigenetic state of the cell.

Indeed, another aspect of the NF-κB DNA-binding response that has been revealed by ChIP-seq experiments is its cell-type- and stimulus-specific nature, with different NF-κB subunits binding to diverse sites under different experimental conditions. For example, Xing et al. (5) compared the genes that were bound and regulated by RelA in TNF-treated HeLa human cervical carcinoma cells, to the direct, transcriptionally regulated target genes identified in LPS-treated U937 and THP-1 human monocytic cells. They found a strikingly small overlap between the sets of genes directly regulated by NF-κB in all three of these scenarios. Although deeper and less stringent analyses of these data may reveal a greater overlap, it is clear that cell type and stimulus combine to regulate chromatin accessibility and focus NF-κB dimer-DNA interactions at a subset of all consensus κB binding sites. Therefore, one role of the very large number of potential κB binding sites may be to allow context-specific and diverse use of the NF-κB pathway in response to a variety of stimuli and across different cell types and states.

Beyond the binding events monitored by ChIP-seq and other protein-DNA interaction assays, the “function” of a binding event is generally assessed by determining the transcriptional outcome of the *nearest* gene. However, this simple view may need to be revisited. Indeed, until recently, it was assumed that the regulatory elements of a gene must be located within several kilobases of its locus, and situated on the same chromosome (73). Contrary to this, there is mounting evidence of functional long-range interactions occurring between genomic regions that are situated megabases apart, and even located on different chromosomes (74, 75). Moreover, chromosome organization studies have implicated RelA-containing NF-κB dimers in the initiation or maintenance of higher-order intra- and inter-chromosomal complexes (76, 77). In particular, Apostolou and Thanos (77) found that RelA-containing NF-κB binding to specialized *Alu* repeats plays an important role in initiating interchromosomal interactions, and in the initiation of the *IFNB1* enhanceosome assembly during the early stages of Sendai virus infection (77, 78). *Alu* repeats are ubiquitous repetitive DNA transposable elements that had been shown to contain putative κB-binding sites; they were later shown to represent 11% of p52-, RelB-, and RelA-bound sites in HeLa cells (1). What becomes clear is that NF-κB dimers, at least RelA-containing dimers, can use long-range intra- and inter-chromosomal interactions to regulate gene expression, meaning that the “nearest gene” method of assessing impact of consensus κB sites likely misestimates the number of functional sites.

Taken together, chromatin state and chromatin organization strongly influence the selection of DNA binding sites by NF-κB dimers and, most likely, the selection of the target genes that are regulated by these protein-DNA interaction events. Analyses that consider binding events in the context of three-dimensional nuclear organization and chromatin composition will be required to generate a more accurate view of the ways in which NF-κB-DNA binding affects gene transcription.

### Competition Between NF-κB Dimers

In addition to cell-state specific chromatin modifications and chromatin conformation, NF-κB-driven transcriptional responses can also be modulated by competition between different dimer species for response element binding (Figure 2). Indeed, while global NF-κB dimer abundance may set the global number of sites that are occupied, which dimers are present pre- and post-stimulus will modulate which sites are transcriptionally activated or repressed, based on relative abundances and affinities. In particular, competition between dimers is consequential when dimers lacking a TD occupy consensus κB sites and limit site availability for newly translocated TD-containing NF-κB dimer binding. As mentioned above, this mechanism of transcription repression has been studied most extensively for the p50 homodimer, which has been shown to play a critical role dampening the inflammatory response [reviewed in (79, 80)]. Specifically, *NFKB1* (p50-encoding) knockout mice have been shown to be more susceptible to several types of infection or infection models [e.g., (81–83)], and some of these responses have been linked to disruption of the transcriptional regulation of inflammatory signals (83, 84). By contrast, perturbations that increase nuclear p50:p50 lead to increased promoter binding by p50:p50 and reduced transcription in response to stimulation of many inflammatory genes (60, 85). Those two examples represent relative extremes of dimer concentrations modulation. Yet, given that, as we discussed above, the nuclear abundances of TD-containing NF-κB dimers appear far from saturating conditions, even moderate changes in nuclear concentration of TD-lacking dimers should affect consensus κB site availability to TD-containing, transcription activating dimer binding (see also Box 1). Taken together, differences in dimer abundances, along with competition for κB sites, help explain why different cell types or states exhibit varied responses to NF-κB-activating stimuli.

### Co-regulators of Transcription

Because DNA binding by NF-κB may not necessarily require high affinity and high specificity [e.g., (3, 22)] and non-NF-κB transcription factors can also bind consensus κB sites due to degeneracies in recognized sequences (86) or as they search for their targets (43, 44), it follows that other transcription factors could act as co-regulators of transcription by competing with or helping recruit NF-κB dimers (Figure 2). In addition, TD-containing NF-κB subunits are also known to interact via their TD with a variety of transcriptional co-factors that modify the chromatin landscape to facilitate NF-κB recruitment and initiate transcription [e.g., (87, 88), and reviewed in (89, 90)]. Here, there are two potential scenarios: (1) the partner transcription regulators pre-exist at the κB sites and activation is rapid, or (2) the partner transcriptional regulators must be activated by the same stimulus that activates NF-κB. In the latter scenario, the time scale of NF-κB's ability to affect transcription will be dependent on the time scale of activation of its co-regulator. If this partner is required for releasing a binding site from a competitor or other barrier to site accessibility, then delayed partner activation is another factor that could explain the delayed NF-κB occupancy at high affinity sites. With these different possibilities, co-regulators could effectively modulate the abundance and identity of available κB sites over time.

How do NF-κB dimers interact with the chromatin modifying machinery? One way is via post-translationally modified NF-κB dimers that are known to recruit the histone acetyltransferases (HATs) p300 and CBP, which promote chromatin accessibility and transcription factor binding. For example, RelA interacts with the CBP/p300 complex once RelA Ser276 has been phosphorylated, following the degradation of IκBα (87, 91). When Ser276 is mutated to alanine to prevent phosphorylation, RelA cannot recruit CBP/p300 and fails to induce transcription at a subset of NF-κB-dependent genes (92). Intriguingly, which genes are affected does not seem directly related to whether they normally show delayed expression or not; some genes whose response normally peaks early show reduced transcription when RelA cannot recruit HATs (92), so there may be additional factors that come into play to determine site accessibility and its timing.

In contrast to RelA, the p50 and p52 NF-κB subunits lack a TD and as such bind to histone deacetylases (HDACs) instead of HATs [e.g., (93)]. HDACs are associated with formation of closed chromatin and transcriptional repression (94). In the context of κB sites found in the HIV genome, the recruitment of HDACs by the p50:p50 homodimer acts to maintain transcriptional repression and latency (93). Upon stimulation with cytokines that activate the NF-κB pathway, RelA-containing NF-κB dimers can displace p50:p50 homodimers and HDACs, leading to restoration of an acetylated histone state and transcriptional reactivation of the HIV genome (93). A similar mechanism has been suggested for the transcriptional regulation of pro-inflammatory cytokine genes in hepatic cells (84, 95). Taken together, these studies show that the interactions of NF-κB dimers with different modifiers of chromatin can result in the dynamic regulation of the chromatin state and of the availability of consensus κB sites for transcriptionally repressive or activating interactions with these dimers.

NF-κB has also been reported to bind cooperatively to many general transcription factors *in vitro*. For example, the *in vitro* assembly of the interferon-β (*IFNB1*) enhanceosome was shown to be dependent upon interactions with the AP-1 family member c-Jun, interferon regulatory factors (IRFs) and activating transcription factor 2 (ATF2) [reviewed in (10) and in (96)]. However, *in vivo* these factors are recruited to the enhanceosome in a stepwise manner, without any dependence upon cooperative interactions (77, 78). As such, there is currently little *in vivo* evidence that NF-κB dimers binding to consensus κB site is enhanced by association with partner transcription factors. Nevertheless, ChIP-seq experiments have detected many instances where NF-κB dimers may be brought to enhancer or promoter sequences lacking κB sites via interactions with another transcription regulator such as PU.1 or ZNF143 (3). Overall, with promoter and enhancer sequences replete with transcription factor binding sites, NF-κB dimers likely partner with other transcription factors to modulate target genes transcription, but how these interactions are coordinated and how they impact transcription is still unclear for most of these targets.

Although here we have only discussed a few specific examples, there are several other points of cross-talk between NF-κB dimers and other families of transcription factors [e.g., nuclear hormone receptors (97) and (33, 98), as well as STATs and IRFs, recently reviewed in (99)]. Overall, it is clear that the differing abilities of NF-κB dimers to recruit other transcriptional regulators via protein-protein interactions and the specific sets of DNA-protein interactions that can take place at each gene's regulatory region could potentially be combined to produce a vast diversity of context-specific, temporally distinct NF-κB-dependent gene expression patterns.

## The Future of NF-κB Research

Understanding the mechanisms that regulate NF-κB-DNA interactions is critical to elucidating the intricacies of NF-κB-specific gene expression profiles. In this review we have discussed the relationships between NF-κB and κB binding sites, and some of the many known complexities of these relationships that affect the regulation of target genes (Table 1). However, despite the wealth of information that has already been uncovered by studies of the NF-κB transcription factors, a comprehensive understanding of the mechanisms underpinning NF-κB-DNA interactions that explain stimulus- and cell type-specific responses remains elusive as numerous questions are yet unanswered.

**Table 1:** Summary of layers of regulations influencing NF-κB-driven gene transcription.

**Quantity**	**Influential factors**
**ABUNDANCE**	
κB binding sites	Number of sites Consensus vs. non-consensus sites Duration and frequency of interactions with DNA Cumulative occupancy of binding sites Avidity vs. decoy site effects of clusters Cooperative vs. independent binding at promoters
Nucleus-localized NF-κB dimers	Homodimerization and heterodimerization Transcription activators vs. repressors Stimulus- and time-dependent changes Competition for subunits in dimerization
**AFFINITY**	
NF-κB dimers for κB sequences	Diversity of bound sequences Dimer-specificity of binding sites Dimer switching and temporal patterns Sequence-specific conformational changes Post-translational modifications of NF-κB subunits
**AVAILABILITY**	
Chromatin state	Histone acetylation and poised chromatin state Cell-type specificity and stimulus-dependence Nearest gene: accessed in 2- vs. 3-dimensions
Competition between NF-κB dimers	Relative abundances Activating vs. repressive dimers Pre- and post-stimulus changes
Co-regulators of transcription	Pre-existing vs. recruited co-regulators HDACs and HATs recruitment Cell-type specificity and stimulus-dependence

*Factors that influence NF-κB-driven gene transcription, its “layers of regulation,” organized by the quantity, abundance, affinity, or availability, with which they were associated in the organization of this review*.

In recent years, our ability to probe chromatin and visualize transcription has considerably advanced, and these advances will be key to developing a better understanding of the complex regulatory processes in the NF-κB system. Chromosome conformation capture (3C) and its subsequent iterations, most recently Hi-C (100), have facilitated the observation of chromatin folding and identification of long-range interactions on a genome-wide scale [reviewed in (101)]. Of particular interest for the study of the interactions of promoters of NF-κB target genes with other regions of the genome, the Promoter Capture Hi-C assay takes promoter-containing fragments from Hi-C libraries and performs paired-end sequencing to identify long-range promoter interactions with distal regulatory elements (102). However, it is important to keep in mind that such methods inform us on the enrichment of particular interactions in bulk populations of cells, an average readout of chromosomal interactions and conformation. Other approaches will be required to understand how different instances of the system vary and how this variability translates into different NF-κB-driven gene expression programs.

Simultaneous developments in the fields of biomolecular labeling and imaging technology have facilitated the visualization of transcription factor dynamics in living cells [reviewed in (29)]. These approaches offer unparalleled insights into the interactions occurring between transcription factors and DNA at the single-cell, single-molecule level. Early studies of transcription factor diffusion and DNA-binding dynamics often used fluorescent proteins and fluorescence recovery after photobleaching (FRAP) assays (29, 103). In FRAP assays, the rate of fluorescence recovery after bleaching then provides information regarding the diffusion and binding kinetics (*k*_on_ and *k*_off_) of a large population of fluorescently labeled molecules (104, 105). However, FRAP measurements fail to accurately capture the heterogeneity in binding dynamics (26, 106).

Single-molecule tracking approaches promise a more complete picture of the different types of dynamic interactions, slow and fast, between NF-κB dimers and DNA. However, two difficult challenges from the use of fluorescent protein tags in these approaches are that the low photostability of fluorescent proteins can severely limit the duration of tracking and the generally high number of expressed fusion proteins yields densely packed, difficult to resolve, transcription factors. The advent of high-brightness, photostable, self-labeling dye tags, relying on fusion with the enzymatic HaloTag and SNAPTag (107, 108) is facilitating long-term imaging of single-molecules at high signal-to-noise ratios. In addition, the development of genome-editing techniques has enabled the tagging of endogenous proteins instead of relying on high-expression exogenous promoters for fusion proteins, thus generating more sparsely labeled populations of molecules to track. Combining these approaches with super-resolution imaging modalities such as photoactivated localization microscopy (PALM) and stochastic optical reconstruction microscopy (STORM) should open the door to the development of a clearer picture of the NF-κB-DNA interactions and subsequent gene expression.

Another quantitative aspect of NF-κB biology that merits a revisit in the future is how the landscape of dimer abundances changes across different cellular contexts and across time. Our current understanding of this landscapes relies mostly on bulk, population-based, endpoint biochemical assays, as well as inferences made from lots of accumulated knowledge from *in vitro* affinity measurements and disruptions of cellular contents with knockout of specific NF-κB subunits [reviewed in (56)]. From many single-cell studies of the dynamics of RelA translocation to the nucleus in different cell types and under different stimuli, we have learned that these dynamics are quite variable [e.g., (109–113)] and, importantly, that the observed variability is absolutely consequential for target gene expression and cellular outcomes [e.g., (14, 69, 72, 111, 114–121)]. However, in all these studies, we are left to assume which RelA-containing NF-κB dimers are actually present in each cell, and each nucleus, and we still lack a similar body of knowledge on NF-κB dimers that do not contain RelA. Capturing live-cell dynamics of the nuclear abundance of other NF-κB subunits and how these dynamics affect transcriptional output of target genes should help us figure out whether what we learned for RelA also applies to these other subunits. Finally, the application of fluorescence correlation spectroscopy (FCS) and fluorescence-lifetime imaging microscopy (FLIM) has begun to reveal aspects of protein dimerization and multimerization in other systems [e.g., p53 tetramerization in irradiated human cells (122) and cell type-determining transcription factors in the *Arabidopsis* root (123)]. In the future, similar approaches should help us broaden our understanding of how NF-κB transcription factors interact with each other, interact with other transcription regulators and interact with DNA in a complex, tunable system that regulates gene expression in many cellular decision processes.

## Conclusions

Over the years, studies have dissected the NF-κB pathway, uncovering many factors and nuances that influence the outcome of DNA binding in this complex system. With hundreds of thousands of DNA binding proteins, and millions of potential DNA binding sites, the recruitment of NF-κB to DNA is regulated in complex ways. This regulation generates gene-, stimulus- and cell type-specific NF-κB responses, allowing NF-κB to respond to numerous different inputs, with a diverse array of outputs. However, a complete, mechanistic understanding of these processes remains unresolved. As we collect better measurements from single-molecule to genome-wide scales, systems biology models may now help us reassemble this dissected system into a framework that can predict ensembles of transcriptional responses.

## Author Contributions

RB, AM, SM, and SG all contributed to the conceptualization, writing and editing of this review. SM performed the simulations presented in the Box 1.

### Conflict of Interest Statement

The authors declare that the research was conducted in the absence of any commercial or financial relationships that could be construed as a potential conflict of interest.
